# Veratrine and Its Use in Surgical Dentistry

**Published:** 1860-07

**Authors:** Leon F. Harvey


### ARTICLE II.

*Veratrine and its Use in Surgical Dentistry.* By LEON F. HARVEY, M. D.

IT is our intention in this article to call the attention of the profession to a remedy, which we presume is but little known or used, and which, if a trial is made of it, will result in much benefit to their patients.

A few remarks as to its origin and its properties may be of interest.

Veratrine is a vegetable alkali, and was discovered by Pelletier and Cavinton, in the year 1819, in the seeds of the veratrum sabadilla, a plant growing in Mexican soil. It has been found in other plants, as the colchicum autumnale, &c., but is obtained mostly from the plant in question. The method of obtaining it from the seeds, we will not detail, being of but little interest to the dental practitioner. In the drug stores we find veratrine, in the form of a light brown or white powder, being entirely inodorous, soluble in alcohol and ether, but sparingly in water. It is poisonous when taken in the mouth; a burning sensation is produced, and it has an acrid taste. It produces intense salivation, and the effect of its application to the nostrils, is a violent fit of sneezing.

We have to do with its external application, and to that our remarks will be confined. Very little irritation is produced by applying it to the skin, though sometimes it blisters. The test of its purity, is a warm and tingling sensation. Until this effect is perceived, Turnbull says, no benefit will arise from the medicine.

The tincture and ointment are used, but having only used the latter, can only speak of that, but writers recommend the ointment as being the best. We were first led to use this article, by Dr. Keep, of Boston. During a visit of his, here, last summer, we had a patient who was suffering intense pain, produced by the plugging of a socket, from which profuse hemorrhage had followed the extraction of a tooth. The doctor advised the use of veratrine, applied to the jaw and cheek. The following preparation was obtained: veratria, gr. xx; glycerine, ʒi; which is the best form to use it, being much pleasanter for the mouth than the common ointment. It was applied, and gave some relief, but not as much as we had hoped for, though the pain could not be entirely removed, it being due to a plug pressed with all the force required to introduce a solid filling. There being great pressure upon the nervous filaments of the parts, medication could not be entirely successful. Determined not to discard it for this failure, it was tried on a patient who was suffering from ulcerated teeth, which she was unwilling to have removed. Here the success claimed for it, by Dr. Keep, was met, to the great joy of the patient and practitioner, for what practitioner is not happy when he has obtained a remedy which will give relief to his patient. Since then we have used it repeatedly in severe cases of ulceration, and been much pleased with the result. Where the teeth give rise to neuralgia, it very often works a cure, that is for the time being. Turnbull speaks of it very highly when used for tic douloureux. He uses veratrine, gr. x to lard, ℥i. A portion the size of a nutmeg is to be rubbed upon the seat of pain, night and morning.

The preparation we used is much stronger, and great care should be observed in its use. Upon the gum, the quantity of the size of a pin's head, can only be used, whilst upon the face, five times that amount. One effect of veratrine is to produce paralysis of the muscles, when too much is used, and, therefore, is to be carefully guarded against.

BUFFALO, *May* 17, 1860.

